# Predictors of cord blood unit cell content in a volume unrestricted large series collections: a chance for a fast and cheap multiparameter selection model

**DOI:** 10.1186/s13287-022-02915-y

**Published:** 2022-06-11

**Authors:** Stefania Fumarola, Alessandra Lucarini, Giovanna Lucchetti, Luana Piroli, Luca Pierelli

**Affiliations:** 1 InScientiaFides Foundation, San Marino, Republic of San Marino; 2 Department of Experimental Medicine, Sapienza University, Rome, Italy

**Keywords:** Umbilical cord blood, CD34+, TNC, Volume, Predictive model, Prenatal and maternal factors, Newborn, Transfusion

## Abstract

**Background:**

Cord blood plays a very important role in stem cell transplantation and therapy with an emerging implication also in regenerative medicine. The number of cells available in a single cord blood unit (CBU), in particular, the CD34+ and total nucleated cell (TNC) content influences the transplantation clinical outcome. We analysed a very large series of CBUs, collected for private banking without any specific volume restriction, to deeply investigate the best predictors of cord blood stem cells content.

**Methods:**

Maternal and neonatal clinic laboratory data of a total 2583 UCBs were obtained from the InScientiaFides cord blood bank based in Republic of San Marino. Univariate and multivariate analysis were conducted to better interpret the data and to build a predictive model to select, the CBU with high CD34+ content.

**Results:**

Our univariate analysis shows that seasonality and the geographical area affects the quality of umbilical cord blood. Gestational age, babie’s gender and birth weight have a positive correlation with CB TNC content. The babie’s birth weight affects positively also CD34+ content and CBU volume while the cesarean delivery affect the CB volume only. Our predictive model, based on multivariate analysis, shows that male babie’s, gestational age lower to 39 weeks, cesarean delivery and CBUs with a content of TNC higher than 3.44 × 10^8^ (group A) have a significant higher CD34+ content than group B (female babie’s, gestational age higher than 39 weeks and vaginal delivery). The group A have a 37.5% of CBUs with a concentration of CD34+  > 2 × 10^6^, while no CBUs with high concentration of CD34+ were detect in group B.

**Conclusion:**

This study, conducted on a very large series of CBUs without any specific volume constraint, highlighted the prenatal and maternal factors that significantly influence the quality of the CBU collected. Specifically, it highlights that volume is not the best predictor of CD34+ CBU content; for this reason it cannot be taken into consideration alone for the analysis of the collected samples. Our final aim is to identify relevant factors, immediately available, that help to choice UCB with high CD34+ cell content, especially in simultaneous deliveries.

## Background

The first cord blood (CB) transplant for hematopoietic reconstitution, occurred 33 years ago in France for a child with Fanconi anemia [1] and represented the groundwork for CB positioning as alternative source of stem cells. Nowadays, CB plays a very important role in stem cell transplantation and cell therapy, with an emerging implication also in regenerative medicine. Recent studies showed how umbilical stem cells exerted therapeutic effects also for the treatment of autism [2], type 1 diabetes [3], acquired sensorineural hearing loss in children [4] and cerebral palsy [5]. Beside the well-known advantages related to the use of CB, this source of hematopoietic stem cells (HSC) contains a higher proportion of primitive HSC, resulting in longer lasting potency and long-term reconstitution [6]. Clinical outcomes after cord blood stem cell transplantation are influenced by the number of cells available in a single cord blood unit (CBU), in particular of CD34+, CD45+ and total nucleated cell (TNC). Some national networks or public banks have adopted a fixed threshold for CB volume to determine the best CBU: most institutions for example discard units with a volume lower than 60 to 80 mL. Actually, is a matter of debate whether the volume of a cord blood is the best predictor for HSC content. In fact, in reports where CB were collected and analysed irrespective of their initial volume, as performed in autologous and family-related context, some smaller collection showed more stem cells than a slightly larger one. For this reason, it is important to widen the analysis of predictive factors to have access to CBU endowed with adequate reconstituting/cellular potential. With respect to TNC content, international institutes adopt a range of CBU eligibility ranging from 5 × 10^8^ to 10 × 10^8^ [7, 8]. In recent years, several private structures have started cord HSC programs of conservation for autologous use (or extended for family use) for future treatments. In this case, the retention policies are less stringent. In unrelated allogeneic setting, recent reports indicate that a dose ranging for 1.0 × 10^5^ to 1.2 × 10^5^/kg CD34+ cells or higher confers to CBU the capacity to reconstitute stable haematopoiesis after transplantation [9, 10]. For autologous CB the safe minimum dose of CD34+ cells is unknown but, due to the lack of histocompatibility diversities, is supposed to be less than that for allogeneic transplantations. Previously, we observed that volume is a less important predictor than TNC for CD34+ cell content and may not be used as a selection criterion [11]. In order to establish a CBU quality, the number of CD34+ and TNC cells in each CBU represent well defined predictors but also maternal and neonatal factors should be evaluated for a more accurate quality prediction of individual CBU, also considering future perspectives where CBU might be used in autologous or family related settings for new therapeutic indications. In this report, we analysed a very large series of CBUs, collected for private banking (autologous and family related), without any specific volume restriction, to deeply investigate the best predictors of stem cells taking also into account some maternal and neonatal factors such as: maternal age, gestational period, mode of delivery (vaginal vs. cesarean delivery), baby’s birth weight, infant’s gender, birth season, geographical area of birth, and child blood group. The present study gives a complete univariate and multivariate analyses of predictors on very large series of CB collection and, differently from the majority of previous studies in this field, is not affected by a preset volume restriction.

## Methods

### CBU selection and collection

For this study, a retrospective analysis of 2583 processed samples was performed from July 2016 to June 2021 (study sample); 180 CBUs collected from July 2021 to December 2021 were analyzed to test a predictive model (test sample). In study sample we assessed volume and cell number during time which were found homogenous considering collections of the first 2 years and of the last 2. The samples were collected in various Italian (birth units) collection points, in both private and public hospitals and subsequently shipped with a dedicated courier for the transport of organs and tissues at a controlled temperature and then processed at the InScientiaFides cord blood bank (CBB) based in Republic of San Marino (accredited FACT-NETCORD on 5 October 2012). All pre and post-natal data, together with those related to the CBU have been made available by the Foundation InScientiaFides (non-profit organization which operates in the Republic of San Marino) that promotes research in the field of stem cell procurement and conservation. All participants provided a written informed consent. Parents were given a kit, complete with all the material necessary for the collection of the CB. The collection kits are produced and packaged in compliance with the Italian Ministry of Health rules for the safety and transport of infected materials and diagnostic samples. The kit components are certified according to directive 93/42/EEC implemented in Italy with legislative decree 46/97. CB collections were performed by trained collection staff using either in-utero or ex-utero collection procedures. At the time of collection, the umbilical cord was stabilized and sterilized with Neoxinal 0.05%; (Nuova Farmec, Settimo di Pescantina (VR), Italy). The umbilical vein was punctured with a 12-gauge needle attached to the collection bag containing 21 mL of CPD anticoagulant (Maco Pharma, Mouvaux, France). The CB was drained by gravity into the collection bag and gently rocked on a rotating scale to ensure adequate mixing. Differences in collection for the two delivery modalities were the following: the collection of the UC in utero involves the positioning of clamps on the UC at a distance of 5 cm from the newborn. The bag was positioned at a lower level to ensure blood flow until it was stopped. Then, the procedure was repeated with the second needle, channeling the umbilical vein at the highest point of the UC, closer to the placenta. The collection of cord blood with placenta ex utero instead involves the removal of the placenta from the uterus and its positioning in a sterile container. The UC was placed downwards to exploit the force of gravity and the cord was punctured at multiple sites using both needles. For both delivery modalities, the UC was repeatedly milked after venipunctures. The sample was placed in the refrigerated kit (Dryce, Milan, Italy), validated for maintenance the temperature between 2 and 8°C for at least 60 h and then sent to the CBB.

The shipping time, on average, was 35 h 60 min, while the entire average processing time was 43 h 56 min, never exceeding 72 h, as required by the Italian law “State-Regions Agreement April 2011” and the FACT-NETCORD provisions.

### CBU processing

The processing of the samples took place in a clean room (Grade B GMP classification) while the collection bag was opened under a sterile hood in laminar flow conditions. All CBUs were processed by manual method which provides the red blood cell depletion and plasma reduction as previously reported by Page et al. 2014 [12]. Briefly, the CBU treatment begins with the isolation of the buffy coat (BC) fraction by transferring the bag content in a suitable number of 50 mL tubes (Euroclone, Pero, Italy) using a 50 mL syringe with a G18 needle and subsequently centrifuged at 3500 rpm for 12 min with acceleration value = 2 and braking value = 0 in a Eppendorf centrifuge 5804 (Eppendorf SE, Hamburg, Germany). After the separation of the components, the plasma was removed with a 10 mL syringe (RAYS S.P.A., Osimo, Italy) and the BC collected using a pasteaur pipette; then, BC was inserted into another 50 mL tube until a volume of 20 mL was reached. TNC and CD34+ were enumerated in the BC to determine final cell count recovery. This processing method allowed the average recovery of 70% TNC, 80% CD34+  and 70% removal of red blood cells RBC. DMSO/Dextran (10% dimethyl sulfoxide in 5% dextran 40, WAK-Chemie Medical GmbH, Steinbach/Ts, Germany) was added as a cryoprotectant and the product was cryopreserved by controlled rate freezing in a 25mL double-compartment cryopreservation bag (Pall Corporation, Covina, CA). Cryopreserved CBUs were stored in air phase of liquid nitrogen.

### CBU TNC count

TNC analyses were performed through an automated haematology system (ADVIA-2120, Siemens Healthcare Diagnostics, Munich, Germany), which is based on peroxidase staining to provide white blood cell (WBCs) counts. Leukocyte count, RBC, hemoglobin (Hb), hematocrit, mean RBC volume, average Hb content in RBC, RBC distribution width, platelets (PLT) count, mean volume of PLT, distribution width of PLT, and nucleated RBC were analyzed. Differential WBC count included lymphocytes, monocytes, neutrophils, eosinophils, basophils and large unstained cells. CBU's TNC counts were obtained by multiplying the absolute WBC counts by CBU volumes.

### CBU CD34+ cell count and viability

Quantification of CD34+ cells was performed with a BD FACSCanto ™ II flow cytometer using reagents of BD Stem Cell Enumeration Kit (BD Biosciences, San Jose, CA). This kit uses a combination of CD45 FITC antibodies (clone 2D1), and CD34 PE (clone 8G12). The assay simultaneously enumerates the total viable dual-positive (CD45+ /CD34+) hematopoietic stem cells giving an absolute count of CD34+ cells/μL and the percentage of viable CD45+ /CD34+ cells. Dead cells were excluded from the count by co-staining with 7-aminoactinomycin-D (BD Biosciences, San Jose, CA).

### AB0 group Rh type confirmation

DiaClon ABD-Confirmation was used for the ABO/RhD blood group control of CB units (DiaMed GmbH,Cressier, France). Brefly, 50 µL of whole blood has been added to 0.5 mL of diluent (DiaMed GmbH, Cressier, France) in a 1.5 mL tube. Subsequently, 12.5 µL of suspension has been withdrawn and pipetted into the microtubes of the ABD cards containing the monoclonal antibody. After centrifugation, (10 min) ABD cards were analysed and blood group determined according to the manufacturer's instructions.

### Statistical analysis

For the statistical analysis of predictors (independent variables) for distinct CD34+ and TNC targets (dependent variables), the age of the mother, the type of birth, the age of gestation (a term pregnancy was considered in the range of 37–41 weeks), the birth weight of the newborn, the volume of CB (intended as the sum of CB volume and anticoagulant), the blood group of the newborn, the geographical area of birth, the birth season and the sex were processed. In a first set of analysis, all continuous and dichotomic independent variables were evaluated. Linear regression and one-way analysis of variance (ANOVA) followed by Tukey’s honest significant difference post hoc test (*p* < 0.05) were used. Two multivariate analysis models were then conducted using the number of CD34+ and TNC as independent variables, against all other dependent variables using multiple linear regression analyses. Moreover, based on multivariate results for CD34+ content, we built a predictive model stratifying the test samples (see “CBU selection and collection”) in two groups: group A (male babie’s, gestational age lower to 39 weeks, cesarean delivery and CBUs with a content of TNC higher than 3.44 × 10^8^; this TNC threshold was chosen considering the median value of the study sample) and group B (female babie’s, gestational age higher than 39 weeks, vaginal delivery and a count of CBUs TNC lower to 3.44 × 10^8^). Data were analyzed using the Student’s t-test to check for a significant difference between groups (*p* < 0.05). All descriptive data were presented as mean, median, range and standard deviation. Univariate and multivariate regression analysis as well as comparisons by one-way ANOVA and Student’s t-test were carried out by Minitab® 16.2 (RMIT University, Melbourne, Australia).

## Results

### CBUs characteristics

The analyses of CBUs collected and processed at InScientiaFides from July 2016 to June 2021 (study sample) demonstrated that 1319 (51.06%) of the CBU collected derived from males, while the remaining 1264 (48.93%) from females. Furthermore, the majority of samples resulted from vaginal 1555 (62.95%) compared to 941 (36.43%) of caesarean deliveries, respectively, with a median birth weight of 3.17 kg (range 1.02–5.04 kg; mean 3.29 ± 1.23). The majority of pregnant women give birth at 39 (median value with a range 30–45; mean 39.09 ± 1.34) weeks of gestation with a median age of 38 years (range 20–58 years, mean 37.56 ± 5.42). The demographic Italian analysis showed that 1054 (40.81%) of the samples come from the north of Italy, while 719 (27.84%), 554 (21.45%), and 256 (9.81%) from the center, south and islands, respectively. In addition, the birth distribution of our series in the various seasons was 600 (23.23%) in winter, 721 (27.91%) in autumn, 632 (24.47%) in spring and 633 (24.51%) in summer (Table 1).

**Table 1:** Clinical characteristics of maternal and infant CBU

	N (%)	Median (range)	Mean (± SD)
Number of CBU collected	2583 (100%)		
Babies’ gender			
Male	1319 (51.06%)		
Female	1264 (48.93%)		
Infant gestational age (weeks)		39 (30–45)	39.09 (± 1.34)
Maternal age (years)		38 (20–58)	37.56 (± 5.42)
Birth weight (kilograms)		3.17 (1.02–5.04)	3.29 (± 1.23)
Type of delivery			
Caesarean	941 (36.43%)		
Vaginal	1555 (62.95%)		
Geographical birth area (Italy)			
South	554 (21.45%)		
Islands	256 (9.91%)		
Center	719 (27.84%)		
North	1054 (40.81%)		
Season of birth			
Spring	632 (24.47%)		
Summer	633 (24.51%)		
Autumn	721 (27.91%)		
Winter	600 (23.23%)		

The clinical characteristics of the samples were the following: the CBUs collected had a median volume of 72 mL (range 15–205 mL; mean 74.46 ± 26.54) with a median time to processing from birth to freezing of 43 h (range 6–127 h; mean 43.67 ± 14.66). After processing, the CBUs median TNC and CD34+ content were 3.44 × 10^8^ (range 2.40 × 10^6^ – 18.83 × 10^9^, mean 3.86 × 10^8^ ± 2.14) and 7.69 × 10^5^ (range 2.40 × 10^3^–1.35 × 10^7^; mean 1.14 × 10^6^ ± 1.18), respectively. Post-process CD34+ viability was, on overage, 95.10% while the analysis of CB blood group showed that 1143 (44.25%) babies had 0 blood group respect to the 954 (36.93%), 280 (10.84%) and 84 (3.25%) A, B, and AB group, respectively (Table 2).

**Table 2:** Characteristics of the processed cord blood units including post-processing cellular content

	N (%)	Mean (± SD)	Median (range)
Number of CBU collected	2583 (100%)		
Collection volume (mL)		74.46 (± 26.54)	72 (15–205)
Time to processing from birth to freezing (hours)		43.67 (± 14.66)	43 (6–127)
CBU TNC count (× 10^8^)		3.86 (± 2.14)	3.44 (0.002–88.3)
CBU CD34+ count (× 10^6^)		1.14 (± 1.18)	0.769 (0.0002–13.5)
CD34^+^ Viability (%)		95.10	
Blood group of babies:			
A	954 (36.93%)		
B	280 (10.84%)		
O	1143 (44.25%)		
AB	84 (3.25%)		

### Post process CBU analyses

**Fig. 1:**
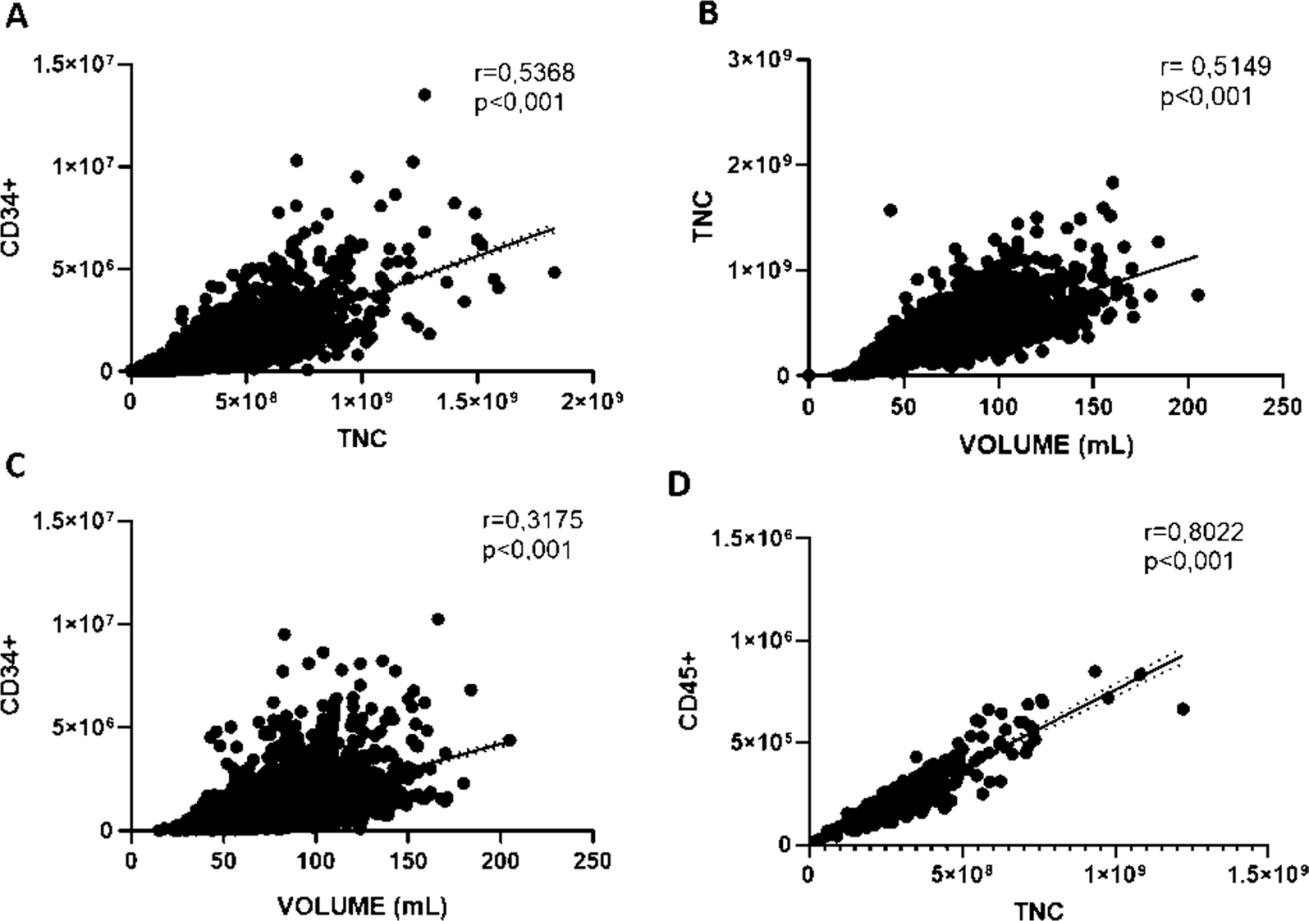
Correlation among the UCB characteristics (N = 2583). Significant positive correlation were observed between (**A**) CD34+ and TNC (r = 0.5368; *p* < 0.001); (**B**) volume and TNC (r = 0.5149; *p* < 0.001); (**C**) volume and CD34+ (r = 0.3175; *p* < 0.001); (**D**) Positive correlation observed among the TNC content and CD45 + (r = 0.8022; *p* < 0.001)

A first set of single-regression analysis strongly confirms in our large-series CBU (study sample) the correlation previously reported by us [11] between CBU TNC and CD34+ (r = 0.5368, *p* < 0.001), TNC and CBU volume (r = 0.5149, *p* < 0.001) and between volume and CD34+ (r = 0.3175, *p* < 0.001). A regression analysis between CD45+ cells and TNC count was performed to confirm the accuracy of our TNC count, showing high correlation with TNC (r = 0.8022, *p* < 0.001) (Fig. 1).

### Maternal and prenatal data analyses

**Fig. 2:**
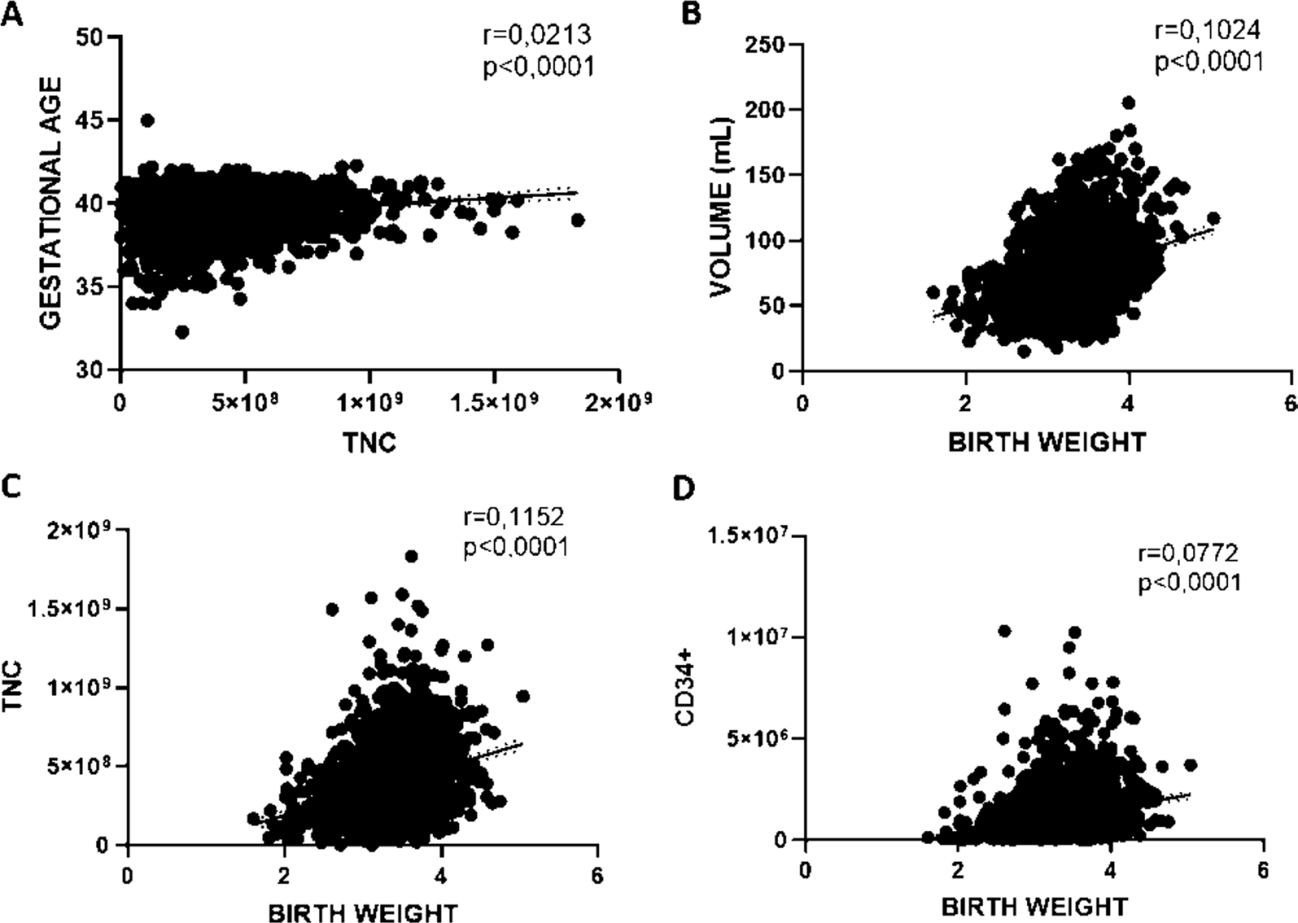
Correlation among the UCB characteristics (N = 2583) and obstetrics and neonatal factors. Significant positive correlation were observed between (**A**) TNC and gestational age (r = 0.0213; *p* < 0.0001); birth weight and volume (**B**) (r = 0.1024; *p* < 0.0001); TNC (**C**) (r = 0.1152; *p* < 0.0001) and CD34+ (**D**) (r = 0.0772; *p* < 0.0001)

Taking into account the gestational age, we found a positive correlation with CBU TNC content (r = 0.0213, *p* = 0.0001) while no correlations were found with CD34+ and volume (r = 0.00008, *p* = 0.65; and r = 0.0008, *p* = 0.15, respectively). Under 39 weeks of gestational age we had an average CD34+ /TNC content of 1.28 × 10^6^/361 × 10^6^, while over 39 weeks we observed a CD34+ /TNC content of 1.1 × 10^6^/401 × 10^6^, respectively (*p* = 0.043 for CD34+ and *p* < 0.0001 for TNC). No correlations were obtained for TNCs and CD34+ content with mother age (r = 0.0006, *p* = 0.198; r = 0.002, *p* = 0.442, respectively) while significant was the correlation with volume (r = 0.0024, *p* = 0.013,) and mother age. Then, we confirmed the correlation between birth weight and CD34+ (r = 0.0772, *p* < 0.005), also showing significant as well as correlation of birth weight with TNC and volume (r = 0.1152, *p* < 0.005 and r = 0.1024, *p* < 0.005, respectively) (Fig. 2).

**Fig. 3:**
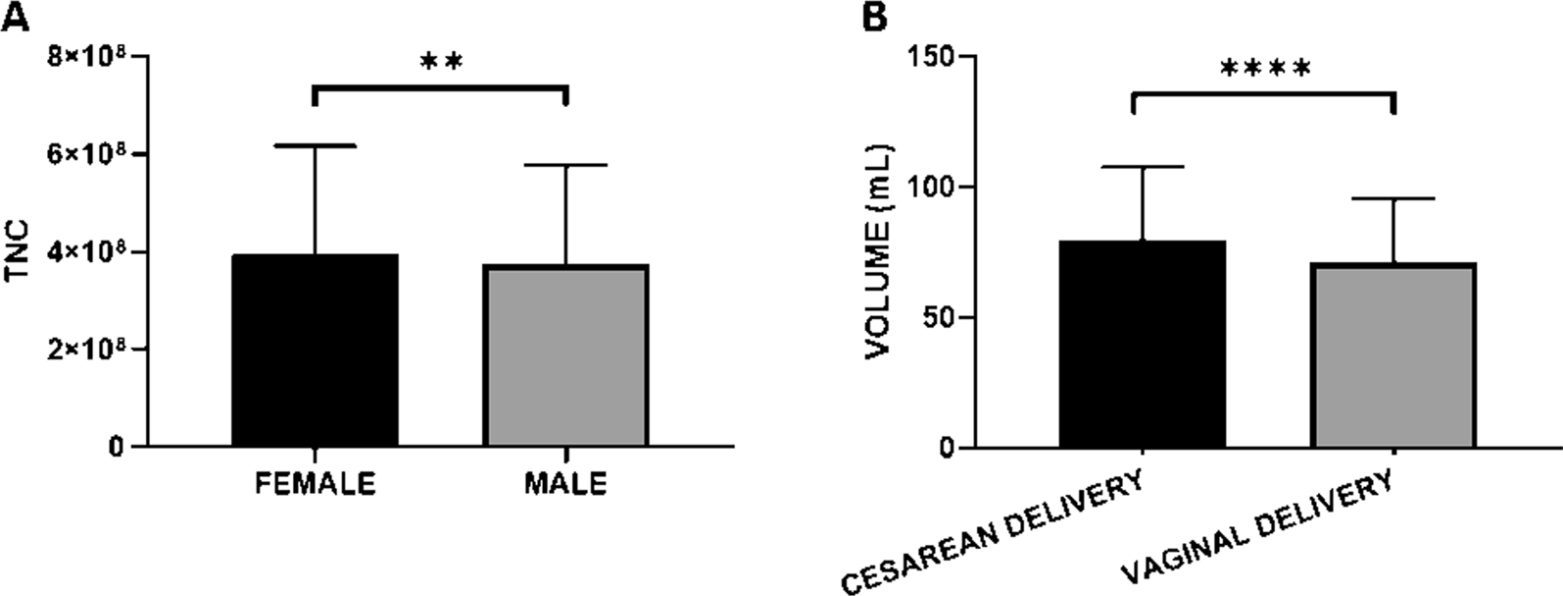
Relation between babies gender and type of delivery with UCB volume and TNC. One-way ANOVA analysis (using Tukey Method post hoc test) showed that child gender affects TNC content (**A**) while birth modality affects the volume (**B**). Particularly females showed a higher TNC content than males (**A**) and the cesarean delivery (**B**) showed more abundant volumes compared to vaginal. All reported values are expressed as mean ± standard deviation (**p < 0.005; **** *p* < 0.0001)

One-way ANOVA analyses showed that child gender affects CBU TNC content while birth modality affects only the CBU volume. Particularly, females (3.97 × 10^8^ ± 2.2 × 10^8^) showed a higher TNC content than males (3.75 × 10^8^ ± 2.2 × 10^8^). Concerning the type of delivery, it influences CBU volume only: cesarean deliveries showed higher volumes (79.76 mL ± 28 mL) compared to vaginal (71.06 mL ± 24.47 mL) (Fig. 3).

The blood group of the child did not show any correlation with sample characteristics considering TNC, CD34+ and CBU volume (Data not shown).

### Seasonal and geographical data analyses

**Fig. 4:**
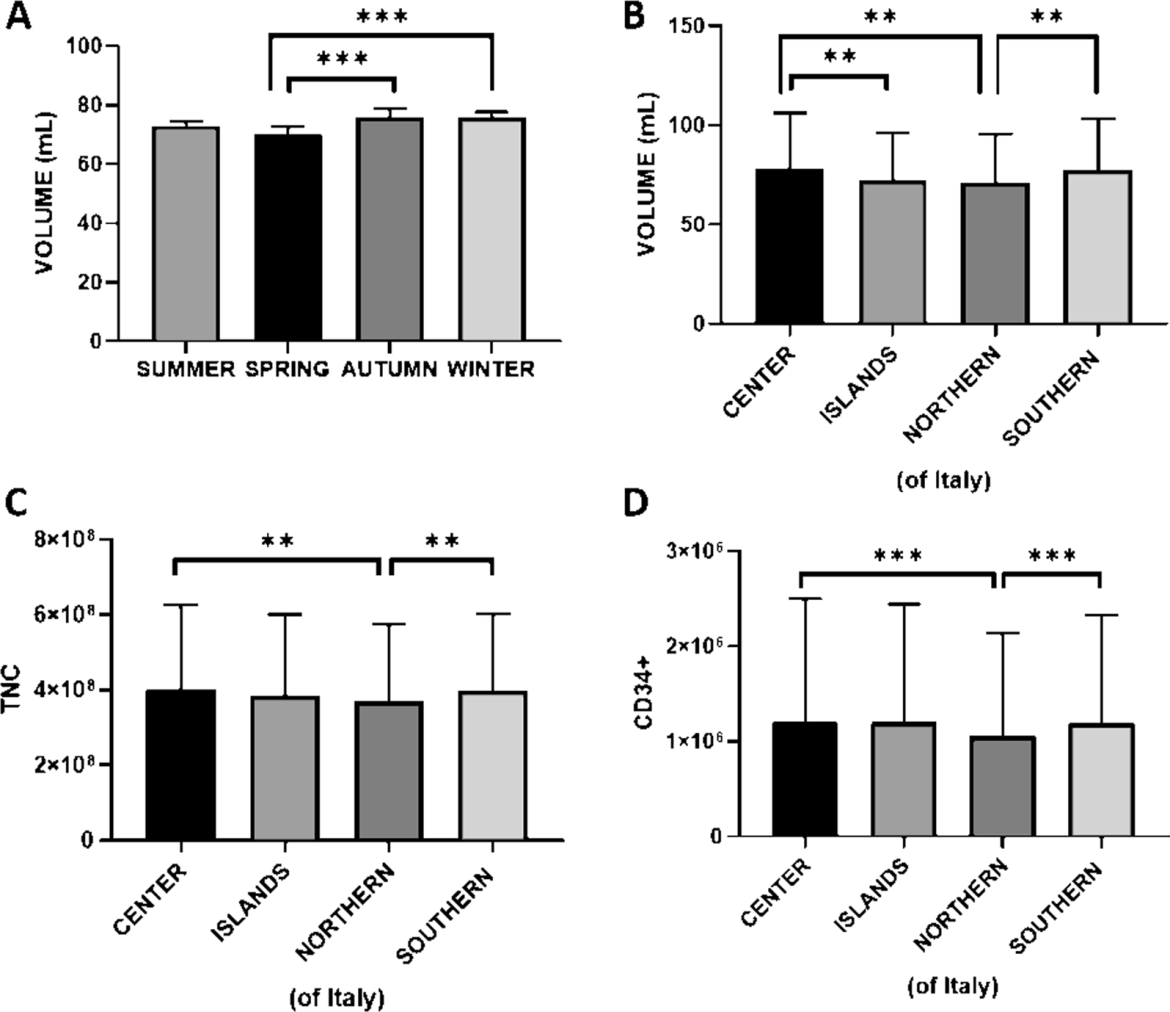
Correlation among the UCB characteristics and seasonal and geographical data. One-way ANOVA analysis (using Tukey Method post hoc test) showed that CBU volume collected in winter and autumn are more abundant than those collected in spring. No significant differences were observed between UCB collected in summer and those collected in other months (**A**). CBU collected in central-southern Italy showed higher volumes (**B**) and higher TNC/CD34+ content (**C**, **D**) than those of northern Italy. For CBU collected in italian islands we did not observe any significant difference as compared to the rest of the country in terms of volume and TNC/CD34+. All reported values are expressed as mean ± standard deviation (**p < 0.005; ***p < 0.001)

The birth seasonal analyses demonstrated that the CBU volume collected in winter and autumn (76.71 mL ± 28.23 mL for winter and 75.63 mL ± 25.84 mL for autumn, on average) are more abundant than those collected in the hot months (71.74 mL ± 24.12 mL for spring, and 73.82 mL ± 26.11 mL for summer, on average) (Fig. 4). No significant differences were observed in TNC and CD34+ amount during the seasonal analysis. Finally, the evaluation of Italian geographical area showed (Fig. 4) that samples collected in the middle-south have higher volumes and higher TNC/CD34+ content than those of north area. For CBU collected in islands, we did not observe any significant difference for TNC and CD34+ counts as compared to the rest of Italy, while lower volumes were observed as compared to CBU collected in the middle-south.

### Multivariate analyses

In order to determine the best predictor of CBU quality, a multivariate analysis was performed taking into account the influence of maternal, pre and postnatal data, as well as geographical and seasonal data. As highlighted in Table 3, the independent variables explain 71% of the variability of the content of TNC (*p* = 0.000).

**Table 3:** Clinical characteristics predictive of TNC content in multiple regression analyses

Donor-related variables	Standardized coefficients β	*t*	*p* value
Sex^a^	− 0.06	− 6.08	**0.000**
Gestational age	0.15	14.01	**0.000**
Delivery method^b^	0.10	8.30	**0.000**
Volume	0.45	35.16	**0.000**
Geografical area^c^	0.00	0.13	0.899
CD34+	0.49	37.74	**0.000**
Season^d^	0.00	− 0.27	0.790
Mother age	0.01	0.71	0.478
Birth weight	− 0.01	− 0.48	0.633

Significant correlation are shown in bold

^a^Female as the reference group

^b^Cesarean delivery as the references group

^c^South as the references group

^d^Winter as the references group

The content of CD34+ (*p* = 0.000) and volume (*p* = 0.000) are the main factors that positively influenced the CB TNC content, as well as delivery method (*p* = 0.000), gestational age (*p* = 0.000) and babie’s gender (*p* = 0.000), (vaginal higher than cesarean, higher gestational age higher than lower, females higher than males). The independent variables considered in multiple regression explain 56% of the variability of the CD34+ content (*p* = 0.000). TNC count represents the most important variable that influenced the CD34+ cells. The gestational period had negative correlation with the CD34+ cell content (*p* = 0.000). Concerning type of delivery (*p* = 0.004) and babie’s gender (*p* = 0.000), we found that cesarean delivery and male infants increase the CD34+ cells content (Table 4).

**Table 4:** Clinical characteristics predictive of CD34+ content in multiple regression analyses

Donor-related variables	Standardized coefficients β	*t*	*p* value
Sex^a^	0.07	5.14	**0.000**
Gestational age	− 0.09	− 6.23	**0.000**
Delivery method^b^	− 0.04	−2.91	**0.004**
Volume	0.02	0.91	0.362
Geografical area^c^	− 0.01	−0.91	0.364
TNC	0.74	37.01	**0.000**
Season^d^	0.00	− 0.21	0.831
Mother age	− 0.02	− 1.32	0.189
Birth weight	0.02	1.48	0.140

Significant correlation are shown in bold

^a^Female as the reference group

^b^Cesarean delivery as the references group

^c^South as the references group

^d^Winter as the references group

In order to validate our results, a predictive model based on results of our multivariate analyses was built using the CBUs data collected from July to December 2021 (test samples; see Materials and Method). *T- student* test results highlighted that CD34+ content of group A (male babie’s, gestational age lower to 39 weeks, cesarean delivery and CBUs with a content of TNC higher than 3.44 × 10^8^) was significant higher than group B (female babie’s, gestational age higher than 39 weeks, vaginal delivery and a count of CBUs TNC lower to 3.44 × 10^8^) (t-value = 7.18; *p* < 0.00001); in addition 37.5% of CBUs had a CD34+ content > 2 × 10^6^ in group A, while no CBUs with a content of CD34+  > 2 × 10^6^ was detected in group B (Table 5).

**Table 5:** Application of the predictive model on the test CBU sample

	Group A (± SD)	Group B (± SD)	*p* value
N°	36	144	–
CD34+ mean (× 10^6^)	1.63 (± 0.78)	0.44 (± 0.27)	< 0.00001
CD34+ median (× 10^6^)	1.54 (0.22–8.09)	0.35 (0.04–1.71)	
Frequency of CB with CD34+ > 2 × 10^6^	37.5%	0%	–

Comparison of CBU of group A (male babie’s, gestational age lower to 39 weeks, cesarean delivery and CBUs with a content of TNC higher than 3.44 × 10^8^) versus group B (female babie’s, gestational age higher than 39 weeks, vaginal delivery and a count of CBUs TNC lower to 3.44 × 10^8^)

Prediction of CD34+ content can also occur when we excluded TNC count from the above mentioned model. Excluding TNC from the model we have access to a cheaper prediction (this prediction may be obtained prior to delivery and avoiding CB collection and TNC count) that, unfortunatly, is characterized by a lower prediction potency (in this case the average CD34+ cell content was 1.12 × 10^6^ for group A and 0.97 × 10^6^ group B respectively; *p* = 0.0459).

## Discussion

To date, the success of a transplant strongly depends on the injected cell dose. CB stem cells have a several strengths: immediate availability, low risk of graft versus host disease and an adequate level of hematopoietic progenitor cells. For these reasons, cord blood represents an alternative source to bone marrow and peripheral stem cells, albeit with a smaller quantity of cells. Investigators suggest some ways to solve this problem: (1) screening and selection cell-rich CB for public banks, (2) choosing the optimal methods to collect, (3) increasing the recovery rate of CB in processing, and (4) promoting an ex vivo expansion of CB stem cells. Numerous studies have been carried out with the aim to identify the factors that influence the cord blood sampling and therefore the content of stem cells. In this retrospective study, we examined a very large series of CBUs, collected in Italy for private banking without any specific volume restriction, to deeply investigate the best predictors of stem cell content, taking into account some maternal and neonatal factors such as: maternal age, gestational period, type of delivery, baby’s birth weight, infant’s gender, birth season, geographical birth area and child blood group. Worldwide, CB banks adopt multiple criteria of selection, including TNC count and volume of collected CBUs. The threshold for volume ranges between 60 and 80 mL: in this way, only CBUs with a higher volume were analysed and enrolled based on the number of TNCs. Actually, higher CB volumes appear as a good predictor of CD34+ cell content in collection series with a volume higher than 80 mL, also in multivariate analysis [12]. However, for a better understanding of independent factors influencing CB TNC/CD34+ cell content in large series, CB volume restrictions or stratifications during analysis could affect or weaken the impact of all analysed variables. In a first set of analyses, we performed univariate linear regression that shows as several maternal, obstetric, and neonatal factors were related to the TNC and CD34+ content of CBU. This data show a significantly higher TNC count in heavier babies. In particular, the increase of one kg of baby weight seems to increase of around 7.66 × 10^7^ CD34+ content, of 19.5 mL volume and of 2.00 × 10^8^ TNC [13, 14]. Like Sparrow et al. [15], we report a significantly higher CB volume in caesarean compared to vaginal deliveries. On the contrary, delivery modality does not influence the content of TNC and CD34+, at variance with univariate results reported by Mohyeddin et al. in a very small series where vaginal deliveries lead to an increase in TNC content [14]. In accordance with our findings, Ballen et al. [16] showed that newborns with longer gestational age had higher TNC counts but lower CD34+ cells at univariate regression analyses. Our univariate data also show that the female babies had more TNC content than male babies. A very slight correlation between mother age and the CBU volume was observed, even though age does not affect the CBU TNC/CD34+ content. In our univariate analyses, the UCB collected in the months of winter and autumn have higher volumes than those collected in spring and summer. We also demonstrated that the samples collected in central-southern Italy have higher CBU volumes and higher TNC/CD34+ content than those of northern Italy, while for CBUs collected in Italian islands no significant differences were observed for TNC and CD34+ content. This phenomenon was then explained by our multivariate analysis that identifies cesarean delivery as significant independent factor: in fact, in south and center of Italy we have observed a significantly higher frequency of cesarean deliveries (data not shown). The relations between obstetric and neonatal factors and CBU cell composition were further investigated on a multiple regression analyses. By the multivariate approach, gestational age, CB volume, babies’ gender and type of delivery show a significant positive influence on the TNC content accordingly to Wen et.al [13]. Recent reports indicate that a dose of 1.0 × 10^5^ or 1.2 × 10^5^/kg CD34+ cells or higher confers to CBU the capacity to reconstitute stable haematopoiesis after transplantation [9, 10]. Hence, considering the relevance of CD34+ for engraftment, we focused our attention on relations between them and obstetric and neonatal factors. In reference to this, male babies, short gestational age and cesarean delivery significantly increase CD34+ UCB content in our multivariate approach. Thus, a predictive model based on these criteria was applied on a second CBU sample (test sample) to assess its ability to select CBU with high CD34+ cell content. As a result, we found that group A of CBU including male babie’s, gestational age lower than 39 weeks, cesarean delivery had a very higher CD34+ content as compared to group B (female babie’s, gestational age higher than 39 weeks and vaginal delivery). A cheaper and immediate prediction may be also obtained excluding TNC from the model (this approach avoids CB collection and TNC count) but with lower performances (see results). Reportedly, Maillacheruvu PF et al. [17] claimed the gravity as positive influencer of blood/CD34+ collection in cesarean deliveries. Moreover, a faster manual extraction of the placenta may reduce the chance of clot formation in UCB [18]. As to the gestational age as predictor, in not so trivial to hypothesize that CD34+ recirculation is higher in younger newborns, as a possible physiological mechanism of stem cells redistribution. Male gender has been identified as a favorable condition for UCB CD34+ cell collection due to a possible influence of a larger haematopoiesis cell mass in males and of genetic mechanisms that regulate to its maturity, size and recirculation of the stem cells pool [19]. Finally, if we refer to unrelated UCB collection where a CD34+ cell content > 2 × 10^6^ is recommended, our data suggest a possible selection approach based on a gestational age < 39 weeks and TNC content > 680 × 10^6^. Combining these selection criteria, the data from our series indicated the possible collection of UCB with a mean CD34+ content of 3.19 × 10^6^ (median 2.77 × 10^6^) with a 70% frequency of UCB with a CD34+ content > 2 × 10^6^.

## Conclusion

This study, conducted on a very large series of CBUs without any specific volume constraint, highlighted the prenatal and maternal factors that significantly influence the quality of the collected UCBs. Specifically, it demonstrated that volume does not represent the best predictor of CBU quality and, for this reason, it cannot be taken into consideration alone for the choice of the CB sample to be collected. Our final aim is to identify relevant factors, immediately available, that help to choice UCB with high CD34+ cell content, especially in simultaneous deliveries. Thus, the best scenario for UCB collection is male baby, a gestational age < 39 weeks, a caesarean delivery and the cheap and fast evaluation of a TNC content high than 3.44 × 10^8^. Limiting discussion to unrelated UCB collection, our data suggest a reasonable strategy to harvest CD34-rich UCB in mother with gestational age < 39 weeks when TNC are > 680 × 10^6^.

## Data Availability

The datasets used and/or analysed during the current study are available on request, by contacting the corresponding author.

## Abbreviations

CBU: Cord blood unit
UC: Umbilical cord
TNC: Total nucleated cell
CB: Cord blood
HSC: Hematopoietic stem cells
CBB: Cord blood bank
BC: Buffy coat
WBC: White blood cell
RBC: Red blood cells
PLT: Platelets
